# A Review of the Physiological Responses and Toxicity Mechanisms of Honey Bees Under Lead Exposure

**DOI:** 10.3390/insects17070709

**Published:** 2026-07-08

**Authors:** Wutao Jiang, Qingxin Meng, Rui Tao, Junjie Zhang, Xueyang Gong, Ruigang Wang, Yakai Tian, Kun Dong

**Affiliations:** 1 Yunnan Provincial Engineering and Research Center for Sustainable Utilization of Honeybee Resources, Eastern Bee Research Institute, College of Animal Science and Technology, Yunnan Agricultural University, Kunming 650201, China; wutaojiang66@gmail.com (W.J.); 13015572330@163.com (Q.M.); 18206750417@163.com (R.T.); 18869634506@139.com (J.Z.); xueyangg11@126.com (X.G.); 2 College of Science, Yunnan Agricultural University, Kunming 650201, China; wrg3767@163.com

**Keywords:** lead (Pb), honey bees, pollution, toxicity

## Abstract

Heavy metal pollution has emerged as a major challenge in global environmental science in the 21st century because of its extensive distribution, long-term environmental persistence, and non-degradability. As indispensable pollinators in ecosystems, honey bees have undergone marked population declines, a trend closely associated with multiple environmental stressors, including heavy metal contamination. Notably, lead (Pb), a widespread legacy toxic metal pollutant, is released into the environment through industrial emissions, vehicle exhaust, and other anthropogenic sources, contributing to long-term environmental contamination. Moreover, Pb can be transferred to honey bees through contaminated pollen and nectar. This exposure route may pose a risk to honey bee health, particularly under chronic or repeated exposure. This review synthesizes current evidence on Pb exposure routes, bioaccumulation, sublethal toxicity, and physiological responses in honey bees, while highlighting major uncertainties in colony-level risk assessment. Pb-associated physiological effects may impair individual bee health, but their long-term implications for colony health require further study.

## Simple Summary

Honey bees may not reliably avoid lead-contaminated food under tested gustatory conditions, which could allow contaminated nectar, pollen, or sugar solutions to enter the colony. Available studies indicate that lead can accumulate mainly in digestive compartments and hive materials, and may affect survival, digestive physiology, antioxidant balance, and neurocognitive performance. However, most evidence remains concentrated at the individual or laboratory level, and the extent to which these effects translate into long-term colony-level impairment requires further validation. This review highlights the need to shift lead risk assessment from acute mortality alone toward chronic, sublethal, and colony-relevant endpoints.

## 1. Introduction

Environmental pollution has become a global challenge because it directly contributes to diseases in organisms across natural ecosystems and can lead to species decline [1]. Since the late nineteenth century, heavy metal pollution has intensified worldwide with the continued expansion of industrial, agricultural, and urban activities, including fossil fuel refining and transportation [2,3,4]. Owing to their persistence and widespread occurrence in the atmosphere, soils, and water bodies, heavy metals tend to accumulate in organisms once they enter the biological cycle [5,6,7]. In contaminated environments, these metals are readily absorbed by plants, resulting in elevated concentrations of elements (e.g., copper (Cu), lead (Pb), and cadmium (Cd)) in crops compared with those under normal conditions, thereby affecting both plant survival and productivity [8,9,10,11]. As pollinators depend on such plants for food, they are consequently subjected to the toxic risks associated with environmental heavy metal contamination. In areas with severe heavy metal pollution, this exposure has been associated with developmental impairment, population decline, and reduced survival in both wild and managed pollinators [12].

Honey bees (particularly *Apis mellifera* and *Apis cerana*) are insects of economic and ecological importance because they produce honey, beeswax, and other hive products and provide essential pollination services for wild plants and agricultural crops. This contribution can be crucial to biodiversity maintenance, sustainable agricultural development, food security, and the continued availability of healthcare-related resources [13,14,15,16]. Accordingly, bee health has attracted considerable attention. Since Colony Collapse Disorder was first recognized in 2006, large-scale colony losses have occurred repeatedly worldwide, giving rise to widespread social concern [17,18,19,20,21,22,23,24]. Although the causes of bee population decline have not been fully resolved, existing studies indicate that the process can be associated with multiple factors, among which heavy metal pollution is regarded as an important contributor [25,26,27,28,29,30,31].

Heavy metals that accumulate in plants can be transferred to nectar and pollen. Subsequently, honey bees are exposed to these metals during floral foraging, which may occur through the consumption of contaminated water, ultimately leading to metal accumulation in the body [32,33]. In addition, honey bees may also be exposed through the inhalation or cuticular adsorption of contaminated air, soil, and plant particles [34,35,36]. Heavy metals with potential toxicity to honey bees include mercury (Hg), Pb, Cd, chromium (Cr), arsenic (As), Cu, nickel (Ni), and zinc (Zn). Notably, Hg, Pb, Cd, Cr, and As are of particular concern because of their pronounced biological toxicity, strong environmental persistence, and marked bioaccumulative potential [27,37]. Certain metals are highly chemically reactive and readily interact with biological macromolecules. Under chronic exposure to toxic metals, or excessive exposure to essential metals, their accumulation within the organism may directly or indirectly compromise biomolecular integrity and disrupt organismal homeostasis [38]. In insects, the detrimental effects of toxic heavy metals are mainly manifested as damage to cellular structures and genetic material, with an impact on individual survival, growth, development, and reproductive capacity [39].

Although substantial progress has been made in heavy metal pollution research, primary knowledge gaps remain in understanding the specific toxicological mechanisms and health effects of different metals in honey bees. Metals (e.g., Hg, Pb, and Cd) may interfere with the nervous, reproductive, and immune systems of bees, thereby inducing individual behavioral abnormalities, reduced fecundity, and population decline. However, the dose–response relationships underlying these effects, their long-term cumulative consequences, and their potential transgenerational risks have yet to be fully elucidated [38,40,41,42,43,44]. Furthermore, honey bees and bee-derived products, including honey, beeswax, and venom, have increasingly been utilized as bioindicators for monitoring environmental toxic metal pollution because of their capacity to bioaccumulate contaminants and their sensitivity as environmental sentinels. This role as “pollution carriers” makes honey bees a critical link between environmental exposure and human health risk. Accordingly, the mechanisms through which they interact with environmental pollutants continue to attract scientific attention [31,37,45,46,47].

Pb is among the most widely distributed legacy toxic metals. Although Hg, Cd, and Cr are also hazardous, Pb has been extensively emitted over a long period due to its use as a gasoline additive, a paint component, and an industrial raw material. Consequently, the concentrations of Pb in soils, water, and air worldwide are significantly higher than the natural background levels [48,49,50]. Because Pb exerts pronounced toxic effects on organisms, including humans, the environmental risks associated with its contamination have attracted broad concerns. Notably, even very low levels of exposure may produce adverse health effects [51]. Pb is naturally widespread and commonly distributed in ores associated with Zn, Pb, and Cu, while also being present in soils, the lithosphere, and sedimentary rocks [52,53,54]. Natural pathways can occur through atmospheric transport, soil movement, forest fires, volcanic activity, soil erosion, and rock weathering [52,55]. As a result, Pb concentrations differ substantially among environmental media and vary with both geographic location and soil characteristics. Current estimates indicate that global atmospheric Pb emissions are approximately 449 million kg per year [56], whereas the median concentration reported for inland waters is 5.81 μg/L [57]. Although globally integrated data on soil Pb levels are limited, most studies have indicated that regional factors can significantly influence these levels [58,59,60].

**Figure 1:**
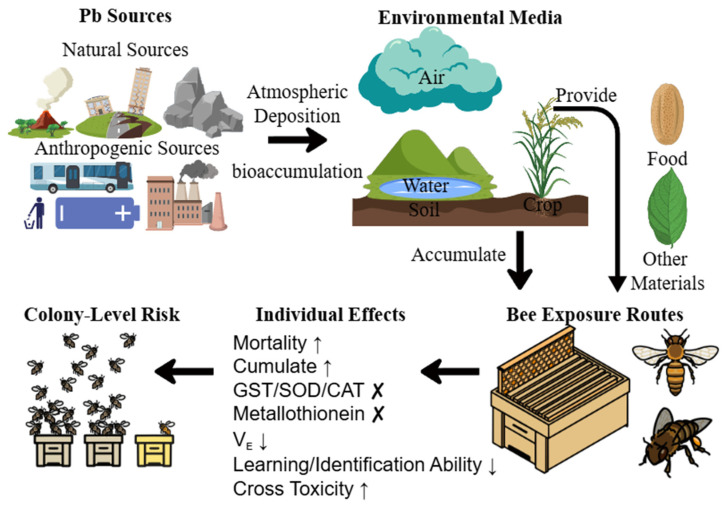
Major sources of Pb emissions, pollution pathways, and impact of Pb on honey bees. Upward and downward arrows indicate increases and decreases, respectively; “×” indicates no consistent or significant response under the reported exposure conditions. Created with BioGDP.com.

Environmental Pb burdens have been markedly amplified by human activities [61,62]. The major sources involve metal mining, smelting, and refining, as well as from fossil-fuel combustion, cement production, phosphate fertilizer use, and the incineration of municipal sewage sludge [63,64,65]. Although Pb has been banned in the electronics industry in most regions because of its toxicity, discarded electronic components remain a continuing source of environmental contamination [66]. Additionally, Pb is extensively used in industrial sectors such as battery manufacturing, fuel smelting, polyvinyl chloride (PVC) stabilizers, colored pigments, and radiation-shielding materials, and these applications continue to release Pb pollutants into the environment [67,68]. Because the physicochemical properties of lead favor both dispersion in natural water systems and atmospheric transport and deposition, Pb contamination in soils is derived from both natural and anthropogenic sources [61,62]. Moreover, owing to its limited mobility within organisms and strong tendency to accumulate in plant and animal tissues, Pb is frequently detected in a wide range of biota, including honey bees [27]. Figure 1 summarizes the principal sources of lead pollution, environmental transport pathways, and the resulting toxic effects on honey bees.

Although several recent reviews have addressed heavy metal pollution in honey bees, Pb deserves a focused synthesis because its risk profile differs from that of metals such as Cd and Cu. Specifically, Pb risk may be underestimated when assessment relies mainly on adult acute mortality endpoints, because available evidence indicates strong digestive retention, bioaccumulation, and possible within-hive persistence. In addition, Pb-related responses appear to involve less consistent activation of classical antioxidant and metallothionein biomarkers, but more evident digestive injury, nutritional antioxidant depletion, and neurocognitive impairment. Finally, most Pb-related evidence remains concentrated at the individual, tissue, or laboratory level, whereas long-term colony-level consequences remain insufficiently demonstrated. This review therefore aims to clarify the specific exposure routes, accumulation patterns, physiological mechanisms, and evidence gaps associated with Pb toxicity in honey bees.

## 2. Exposure Pathways, Uptake Routes, and Spatiotemporal Heterogeneity of Pb Exposure in Honey Bees

Environmental Pb, to which bees are exposed, enters the body mainly through two principal routes. Overall, dietary ingestion and external particle contact are two major routes by which lead is introduced into bees and subsequently exerts toxic effects.

### 2.1. Exposure Through the Food Chain and Subsequent Flower-Visiting Behavior of Honey Bees

For many insects, dietary ingestion is a major route of metal entry, and the midgut epithelium represents the first major interface for soluble metal uptake [69,70,71,72,73,74]. In honey bees, Pb exposure occurs predominantly through the collection of contaminated nectar and pollen from plants growing in polluted environments [27]. Plants can absorb and accumulate heavy metals from contaminated soil and air, after which these contaminants are translocated to the nectar and pollen they produce. Because pollen and nectar are essential food resources for bees, they have been identified in multiple studies as major vectors of lead exposure [75].

Although certain studies have suggested that bees may lack the sensory capacity to directly detect heavy metal contamination in floral resources [76,77,78], Pb exposure can still indirectly alter foraging behavior through the sublethal effects it induces. After consuming contaminated food, bees may experience physiological stress or neurological dysfunction, which can affect their behavioral performance. These sublethal effects may manifest as lower visitation frequencies to contaminated flowers, shorter handling times per flower, shifts in foraging preference, and an overall reduction in foraging activity [76,77,78]. Field observations have demonstrated that bees can reduce their visits to sunflowers grown in Pb-contaminated soil, with significantly shorter foraging durations recorded on individual flowers [79]. Such behavioral changes might contribute to reduced foraging efficiency and, under chronic or high-exposure conditions, could add to the energetic challenges faced by colonies.

Although bees can avoid certain warning colors, insecticides, and bitter compounds, their ability to detect Pb^2+^ through gustation is limited. Proboscis extension reflex (PER) assays indicate that honey bees may not reliably discriminate sucrose solutions containing Pb ions at tested sublethal concentrations, suggesting limited gustatory avoidance under these experimental conditions [77]. This result reveals that exposure to Pb in contaminated environments is largely passive because lead ions did not elicit detectable gustatory avoidance in PER assays. Consequently, foraging bees may fail to avoid Pb-contaminated sugar solutions or floral resources under some exposure conditions, allowing Pb to be transported into the colony with food resources [77].

### 2.2. Exposure via Air and Contact, and Post-Exposure Grooming Behavior in Honey Bees

In addition to dietary exposure, honey bees may also be exposed to airborne Pb-containing particles through inhalation and cuticular contact [80,81,82]. Studies have reported that bees collected from industrially polluted areas with high levels of atmospheric contamination often carry particulate matter containing Pb and other heavy metals on their body surfaces. Therefore, the adhesion of these particles to the bee exterior constitutes a direct route of exposure.

Compared with oral intake, Pb exposure through cuticular contact and inhalation may present lower bioavailability. Research has indicated that when relatively high concentrations of Pb are detected in particles attached to the outer surface of bees, the corresponding concentrations in the hemolymph remain low [27,83]. This finding suggests that only a small fraction of externally adhered Pb is ultimately internalized through autogrooming, while the digestive tract is likely to remain the main site of Pb absorption and accumulation [27]. Nevertheless, high atmospheric Pb levels remain a persistent environmental stressor and may further increase oral exposure by direct deposition onto plant surfaces, such as flowers.

### 2.3. Spatiotemporal Differences in Pb Exposure of Honey Bees

Patterns of lead exposure in honey bees are complex, largely because Pb pollution exhibits pronounced temporal, spatial, and geographical variations [27,75,83,84,85]. This complexity reflects not only the heterogeneous distribution of environmental Pb contamination but also the dynamic interaction between bees and contaminated surroundings. Spatial differences in honey bee Pb exposure are closely associated with the distribution of local flora [86]. Different plant species, as well as cultivars or genotypes within the same species, can demonstrate significant differences in their capacity to absorb and accumulate Pb [52]. Such variations add uncertainty to exposure assessments. However, data on Pb concentrations in the pollen and nectar of different plant species remain limited, which restricts the accuracy of spatial risk evaluation of Pb exposure in honey bees.

From a geographical perspective, the Pb burden in honey bees is closely related to natural and anthropogenic emission sources surrounding apiaries. Significant Pb accumulation has been detected in bees from industrial zones, urban areas, agricultural regions, and rural sites worldwide [32,37]. Available evidence indicates that Pb pollution is most severe in industrial and mining areas, followed by urban regions, whereas contamination is common in seemingly unpolluted rural environments. This geographical heterogeneity can contribute to substantial differences in the exposure pressure experienced by honey bee colonies across different regions [35,45,83,84,85].

The internal Pb content of honey bees can vary by more than five-fold across different regions. Furthermore, Pb levels in honey bees can exhibit evident temporal variability, and fluctuations have been reported even within the same study area. In winter, increased fossil fuel combustion leads to higher Pb emissions. In spring and summer, bees are more active and have more opportunities to contact pollution sources. As a result, environmental Pb levels vary continuously across seasons [27,84,85]. Pb pollution may vary markedly over short timescales [87]. In densely populated and industrialized regions, the local exposure pressure on bee populations can be influenced by changes in anthropogenic Pb emissions resulting from adjustments in industrial activity or traffic regulation.

Overall, Pb exposure in honey bees occurs mainly through the ingestion of contaminated pollen and nectar, a pathway shaped by both Pb accumulation in plants and exposure-related changes in bee foraging behavior. Airborne Pb-containing particulate matter provides a direct route of external exposure and contributes indirectly to oral intake through deposition onto floral surfaces. These pathways determine Pb accumulation in individual bees and across the colony. Accordingly, Pb exposure in honey bees is characterized by pronounced spatiotemporal heterogeneity and dynamic variation. This pattern reflects not only the combined effects of natural and anthropogenic Pb sources but also the complex interactions between bees and contaminated environments.

## 3. Toxic Effects and Physiological Mechanisms of Pb in Bees

### 3.1. Acute Toxicity, Bioaccumulation, and Within-Hive Retention of Pb in Honey Bees

Although direct evidence of the specific effects of Pb on honey bee survival remains limited, available studies indicate that Pb can exert both lethal and sublethal effects on larvae and adults. Di et al. evaluated the acute toxicity of Pb, Cd, and Cu to honey bee larvae and foragers under laboratory conditions [42]. Similarly, Schmarsow et al. reported that the median lethal concentration (LC50) of Pb in forager bees was 345 mg/L, which was substantially higher than the corresponding LC50 values for Cd (78 mg/L and Cu (72 mg/L) [88]. In contrast, honey bee larvae demonstrated greater sensitivity to Pb, with an LC_50_ of 1.12 mg L^−1^. Although this value remained higher than that reported for the more toxic Cd (0.275 mg L^−1^), it fell within the concentration range that may occur in polluted environments [42]. These findings indicate that the acute lethal toxicity of Pb in adult bees is lower than that of Cd and Cu. Its ecological risk may be underestimated in conventional assessments that place greater emphasis on acute lethal endpoints.

The principal risk of Pb to honey bees lies in its high bioaccumulative capacity and the subsequent range of sublethal effects. Di et al. suggested that the Pb burden in both larvae and adults increased linearly with dietary Pb concentration, confirming the substantial bioaccumulation at the individual level [42]. In foraging workers, internal Pb accumulation exceeded control levels by more than 100-fold when food containing 400 mg/L Pb was collected. Using precise organ dissection and graphite furnace atomic absorption spectrometry (GFAAS), Raes et al. found that Pb in honey bees was mainly concentrated in the digestive system [89]. The midgut (including Malpighian tubules) accounted for 67.03% of total body Pb, with a dry weight concentration of 1092 μg/g. The rectum accounted for 27.13%, with a dry weight concentration of 68.1 μg/g. Collectively, these two compartments contained more than 94% of total body Pb. In contrast, the head, thorax, and abdomen (without internal organs) accounted for only 0.45%, 2.42%, and 2.97% of total Pb, respectively. These results suggest that Pb is largely retained within the digestive tract and has limited translocation to other tissues. This conclusion is consistent with a large number of subsequent research findings [90,91,92,93,94,95].

Raes et al. further revealed the temporal dynamics of Pb accumulation [89]. During the larval and early adult stages, bees primarily consume pollen, and Pb accumulation is slow. After transitioning to foraging bees, their sugar syrup intake increases, causing Pb levels to rise sharply. Accumulation reaches a plateau around day 26. Furthermore, Pb elimination is extremely slow. After 12 days without exposure, only one-third of the initial Pb load was cleared, indicating the strong persistence of Pb in honey bees. Additionally, under small-cage laboratory conditions, bees cannot defecate normally. As a result, feces (containing Pb particles) accumulate in the rectum. Therefore, Raes et al. [89] speculated that rectal Pb levels in field-collected foragers are likely significantly lower than those measured under laboratory conditions.

Dabour et al. further confirmed the deposition and localization of Pb in critical tissues through direct detection of Pb signals in the midgut epithelium [96]. Hladun et al. indicated that Pb transported to the hive by foragers gradually accumulated in nest materials (e.g., bee bread and beeswax), thereby creating a potential reservoir for repeated or chronic exposure within the colony [97]. Such retention may increase the possibility of exposure across successive brood-rearing cycles, but direct evidence for transgenerational toxicity remains limited. In addition, a series of studies by Hu Meng et al. indicated that Pb accumulated progressively in the comb and cocoon silk of *Apis cerana cerana* with prolonged comb use [98,99,100,101]. After eight generations of rearing, the Pb concentration in cocoon silk reached 1.131 mg/kg. This value was 2.12-fold higher than that observed after a single generation (0.534 mg/kg). The difference was statistically significant (*p* = 0.0053). Although this species may reduce exposure to some extent by gnawing away old combs, this behavior provides only limited protection because it cannot interrupt the continued input of heavy metals.

Forager bees cannot prevent pollutants from entering the colony at the source. Even if old comb is gnawed as a self-protective response, the overall Pb burden in the hive materials continues to accumulate. This phenomenon highlights both the concealed and persistent nature of Pb pollution. Although the acute toxicity of Pb is relatively low, its ecological risks should not be underestimated. The accumulation of Pb in wax, bee bread, comb, and cocoon silk indicates that hive materials may act as a reservoir for chronic exposure, but the magnitude of resulting colony-level impairment remains insufficiently quantified.

### 3.2. Pb-Specific Physiological Responses in Comparison with Cd Toxicity

Internal Pb accumulation does not necessarily predict the magnitude or type of toxic effects. Therefore, this section examines Pb-specific physiological responses in honey bees, using Cd toxicity as a comparative reference where available. To clarify the Pb-specific contribution of this review, the main toxicological features of Pb exposure are compared with those of Cd exposure in honey bees (Table 1).

Cd toxicity provides a useful comparative framework for clarifying Pb-specific responses in honey bees because Cd has been more extensively discussed in recent reviews of metal toxicity in bees. In general, Cd exposure has been more consistently associated with metallothionein induction, antioxidant enzyme responses, calcium-related disruption, and neuromuscular or developmental impairment [102]. By contrast, the available evidence for Pb in honey bees suggests a different and a less consistently characterized toxicological profile. Pb appears to be characterized by strong digestive retention, limited gustatory avoidance under tested conditions, inconsistent activation of classical antioxidant and metallothionein-mediated detoxification pathways, depletion of nutritional antioxidants such as vitamin E, and impairment of learning- and memory-related behaviors. Therefore, comparison with Cd helps clarify why Pb warrants a focused synthesis: its risk may be driven less by acute lethality or robust detoxification-gene induction and more by chronic retention, digestive injury, and neurocognitive disruption.

Although field studies have suggested that Pb may affect the antioxidant and detoxification systems of honey bees, these observations are complicated by environmental confounding factors [41,91,103]. As a non-redox-active metal, Pb is generally thought to induce oxidative stress by binding to sulfhydryl groups [104]. In honey bees, the action appears to differ from that of other heavy metals. Detoxification in bees mainly relies on antioxidant defenses and detoxification enzymes [78,105]. Notably, the glutathione S-transferase (GST) family is a key component, and many *GST* genes are readily induced by insecticides, pathogens, and heavy metals [106]. In comparative laboratory assays, Pb did not induce the same SOD, CAT, or GST responses observed after Cd or Cu exposure, suggesting that Pb-induced oxidative imbalance may not be adequately captured by classical enzymatic biomarkers alone [38,40,103]. This does not indicate that Pb lacks oxidative toxicity; rather, it suggests that Pb may differ from Cd in the way it perturbs redox homeostasis and detoxification signaling under the tested conditions. In addition, Meng et al. reported that heavy metal pollution, including Pb, induces high expression of detoxification genes, such as *AccCYP4AV1*, *AccCYP314A1*, *AccCYP4AZ1*, and *AccCYP6AS5*, in *Apis cerana cerana* [99]. However, it remains unclear whether Pb exposure directly causes upregulation.

A comparable pattern has been reported for metallothioneins (MT), which are the major metal-chelating proteins in organisms [107]. In the study by Purać et al., MT expression was significantly upregulated after Cd and Cu exposure but was not significantly altered after Pb exposure, indicating that Pb may differ from Cd in its capacity to activate MT-mediated detoxification under the tested conditions [43]. Another study showed that low-dose Pb exposure significantly reduced vitamin E levels in honey bees [108]. As vitamin E is a major lipid-soluble antioxidant, its depletion can increase the susceptibility of cell membranes to lipid peroxidation.

More direct evidence has been obtained from cellular and tissue-level observations. Transmission electron microscopy by Dabour et al. revealed typical lesions in the midgut epithelial cells of Pb-exposed bees, including chromatin condensation, microvillar disruption and atrophy, and mitochondrial swelling [96]. Schmarsow et al. reported that Pb exposure significantly reduced the activity of key digestive enzymes in honey bees [88]. Thus, Pb impairs both the structure and function of the digestive system. Consequently, nutrient digestion and absorption are hindered, providing a direct pathological basis for delayed development, reduced body weight, and energy deficiency in bees.

Taken together, these studies suggest that Pb differs from Cd in both toxicokinetic and toxicodynamic patterns in honey bees. Whereas Cd-related responses are often interpreted through metal-binding proteins and antioxidant enzyme activation, Pb toxicity appears to involve digestive retention, epithelial injury, nutritional antioxidant depletion, and neurophysiological disruption, with less consistent induction of classical GST-, SOD-, CAT-, and MT-related responses. This comparison should not be interpreted as evidence that Pb bypasses all detoxification pathways. Rather, it indicates that currently available biomarkers may capture Pb toxicity less effectively than Cd toxicity, and that tissue-specific and long-term low-dose studies are needed to define Pb-specific mechanisms more precisely.

### 3.3. Neurocognitive Toxicity of Pb in Honey Bees

The biochemical toxicity described above is even more pronounced in another critical physiological system of honey bees: the nervous system. Recent behavioral ecology studies have indicated that Pb impairs the neurocognitive functions of honey bees. This effect likely represents a major component of its sublethal toxicity.

Lead affects the nervous system and behavior of honey bees by disrupting neurochemical processes. Studies have indicated that Pb exerts a bidirectional effect on acetylcholinesterase activity in bees, inhibiting the enzyme at low concentrations (0.1 mg/L) but enhancing its activity at high concentrations (10 mg/L). Such disturbances in acetylcholine hydrolysis directly disrupt normal neuronal signal transmission [38]. Acute exposure to lead slows down appetitive learning and disrupts 24 h memory retrieval in honey bees [44]. Furthermore, chronic exposure to trace lead (0.75 mg/L) leads to significant bioaccumulation (0.809 ± 0.044 mg/kg dry mass) and specifically impairs reversal learning, indicating a loss of cognitive flexibility [109].

Pb can affect feeding-related behaviors in honey bees shortly after exposure, but the response appears to be complex rather than simply inhibitory. Acute sublethal Pb exposure altered feeding-related responses in a concentration-, assay-, and stimulation-site-dependent manner, but the response was not simply linear across concentrations [76]. Under field conditions, soil Pb contamination has also been associated with altered bee foraging behavior, as bees spent less time visiting sunflower heads grown in Pb-contaminated soil, although visit frequency was not significantly affected [79].

In summary, Pb exposure has been consistently associated with impaired olfactory learning, memory retention, and other cognitive functions in honey bees under laboratory conditions. These behavioral deficits indicate that the nervous system is an important target of Pb toxicity. However, direct evidence linking Pb exposure to specific neuroanatomical alterations in the honey bee brain remains limited. Field investigations conducted in areas contaminated by multiple trace metals (predominantly As together with Pb, Cd, Cu, Ni and Zn) found that bees exhibited significantly reduced antennal lobe volume, suggesting that chronic environmental metal exposure may affect neural structures involved in olfactory processing [110]. Nevertheless, because these investigations involved mixed-metal exposure, the specific contribution of Pb cannot be distinguished. Future studies combining controlled Pb exposure with neuroanatomical and neurophysiological analyses are therefore needed to establish whether Pb alone induces structural remodeling of the honey bee brain.

### 3.4. Potential Colony-Level Consequences of Chronic Pb Exposure

Individual-level effects of Pb exposure may have colony-level relevance, but the strength of this link remains insufficiently quantified. Pb-induced changes in survival, feeding responsiveness, learning performance, digestive function, and foraging behavior could reduce the efficiency of foragers or nurse bees under chronic exposure conditions. In addition, Pb retained in bee bread, beeswax, combs, or cocoon silk may serve as a potential reservoir for repeated exposure across brood-rearing cycles. This section examines how Pb toxicity at the individual level is ultimately amplified into the risk of colony-level stress through the division of labor and compensatory mechanisms within a colony.

Through multiple toxic mechanisms, Pb exposure can ultimately initiate a progressive deterioration process in honey bee colonies, extending from individual mortality to possible colony-level vulnerability. A decline in the foraging capacity of individual bees not only reduces the colony’s ability to acquire energy but also contributes to a series of downstream effects. Although no significant difference in sealed brood area was detected during the experimental period, Pb accumulation in bees and hive materials suggests a potential reservoir of chronic exposure that could become more relevant over longer timescales or under additional stressors [97]. This result indicates that Pb can accumulate within colonies in a concealed manner. With increasing rearing cycles, such accumulation may eventually reduce sealed brood area and lower larval survival. Di et al. further indicated that the median lethal concentration (LC_50_) of Pb for larvae was relatively low (1.12 mg L^−1^), revealing that Pb exposure could directly increase the larval mortality [42]. This high sensitivity during early development can severely affect the colony reproductive success. Schmarsow et al. showed that the chronic Pb exposure significantly shortened the lifespan of adult worker bees. In addition, the associated reduction in foraging performance can further weaken colony resilience to environmental stress [88].

These findings indicate that without immediate mass mortality, the worker population within a colony may decline at an accelerated rate. The progression from reduced larval numbers to rapid loss of adult workers and weakened colony resistance to stress illustrates the toxicological nature of Pb. Rather than causing only direct mortality, Pb progressively erodes the dynamic homeostasis and adaptive capacity required for colony persistence.

### 3.5. Combined Toxic Effects of Multiple Pollutants on Bees

Most studies mentioned above were conducted under single-Pb exposure in laboratory or semi-field conditions. However, in real agricultural and industrial pollution environments, Pb typically coexists with other pollutants, forming complex contamination systems. Interactions between Pb and co-occurring pollutants may accelerate, delay, or alter toxic effects. Understanding combined toxicity is essential for accurately assessing the risks to field colonies.

The interaction effects of multiple pollutants can be divided into three categories based on the strength of their combined actions [111]. Synergistic effects occur when the combined toxicity exceeds the sum of individual toxicities. Antagonistic effects occur when the combined toxicity is lower than the sum. Additive effects occur when the combined toxicity equals the sum of individual effects.

Under natural conditions, Pb rarely occurs alone. In contrast, it often co-occurs with other pollutants, forming complex contamination mixtures that affect honey bee health through synergistic or antagonistic interactions. Xue et al. showed that when Pb ions were combined with the widely used herbicide glyphosate, a significant synergistic toxic effect on bees was produced [112]. Under these conditions, the toxicity of the mixture exceeded the sum of the toxicities of the individual components. A more complex interaction pattern was reported by Naggar et al. When highly toxic cadmium oxide nanoparticles (CdO NPs) were co-administered with less toxic lead oxide nanoparticles (PbO NPs), the combined effect was antagonistic, and the overall toxicity of the mixture was lower than that of CdO NPs alone [113]. This result may be associated with differences in nanoparticle surface properties, particle size distribution, and metabolic processing within the organism, highlighting the complexity of pollutant interaction mechanisms.

Importantly, the combined effects of multiple pollutants on bees are not consistently adverse. Compared with exposure to Pb alone, combined treatment with Pb and Zn partially reduced Pb toxicity [114]. This mitigating effect may be attributed to the antagonistic action of Zn against Pb, including competitive inhibition of Pb uptake, enhanced Pb excretion, and modulation of Pb toxicity pathways. Overall, these findings indicate that the toxicity of Pb under natural conditions strongly depends on the type, concentration, and interaction mode of co-occurring pollutants. This complexity in combined toxicity not only increases the uncertainty of bee health risk assessment but also highlights the need for coordinated management of multi-pollutant contamination.

## 4. Conclusions

This review synthesizes the available evidence to show that Pb may act as a chronic and relatively concealed stressor in honey bees. Its effects appear to involve limited gustatory avoidance, digestive retention, tissue injury, altered antioxidant balance and neurocognitive impairment.

At the individual level, these physiological and neurobehavioral effects have been associated with shortened lifespan, reduced foraging motivation, and lower foraging efficiency. At the colony level, these individual effects could contribute to reduced foraging performance or brood-rearing capacity, but direct long-term colony-level evidence remains limited. Under real multi-pollutant conditions, the risk posed by Pb is more complex. Pb may act synergistically with pesticides, such as glyphosate, and may also participate in unpredictable interactions with other metal-based nanoparticles. These combined effects indicate that traditional risk assessment frameworks based on single pollutants and acute lethal endpoints are insufficient to capture the chronic stress and ecological complexity associated with lead pollution in honey bees.

Overall, Pb may represent a chronic and concealed stressor for honey bee health. Addressing this challenge requires a substantial shift in ecotoxicological research and practices. Priority should be given to three transitions: from lethal to sublethal endpoints, from single-pollutant testing to combined exposure assessment, and from the protection of individual survival to the protection of overall colony health. Such shifts will improve the capacity to prevent and manage the chronic ecological risks posed by lead pollution and, in turn, may improve the ecological relevance of Pb risk assessment for pollinator health.

## Figures and Tables

**Table 1:** Comparative toxicological features of Pb and Cd exposure in honey bees.

Toxicological Aspect	Cd Exposure	Pb Exposure	Pb-Specific Implication
General toxicity	High acute and sublethal toxicity	Lower adult acute toxicity, but strong accumulation	Acute mortality may underestimate Pb risk
Main target pattern	Systemic physiological disruption	Digestive retention and gut injury	Midgut may be a key Pb target
Antioxidant response	More evident SOD, CAT, and GST responses	Weaker or inconsistent SOD, CAT, and GST responses	Classical enzyme markers may be insufficient
Metallothionein response	Clearer MT induction	MT not significantly induced in some studies	Pb may differ from Cd in MT-mediated detoxification
Cellular and nutritional effects	Oxidative stress and developmental impairment	Vitamin E depletion, epithelial damage, reduced digestive enzymes	Pb may impair digestion and nutrient balance
Neurobehavioral effects	Neuromuscular and calcium-related disruption	Impaired feeding response, learning, memory, and cognitive flexibility	Neurocognition is an important Pb endpoint
Colony-level relevance	Inferred from survival and development effects	Inferred from chronic retention, foraging impairment, and lifespan reduction	Long-term colony evidence remains limited

## Data Availability

No new data were created or analyzed in this study. Data sharing is not applicable to this article.
